# Microwave-induced hyperthermia in situ in the treatment of tumors of proximal humerus: long-term results with functionary sparing surgery

**DOI:** 10.1186/s13018-023-03895-2

**Published:** 2023-06-13

**Authors:** Cheng-gang Pang, Zhi-fa Huang, Shao-lin Ji, Hong Zhang, Yun-long Zhao, Yong-cheng Hu

**Affiliations:** 1 Department of Orthopedic Surgery, Zhoucheng People’s Hospital, Jining City, 273500 Shandong Province China; 2 Department of Neurosurgery, Tianjin Huanhu Hospital, No.6 Jizhao Road, Jinnan District, Tianjin, 300350 China; 3 Department of Trauma and Hand-Foot Surgery, Shandong Provincial Third Hospital, Shandong University, Jinan, China; 4 Department of Orthopedic Oncology, Tianjin Hospital, No. 406, Southern Jiefang Road, Hexi District, Tianjin, 300211 China; 5 Department of Rehabilitation, Tianjin Hospital, No. 406, Southern Jiefang Road, Hexi District, Tianjin, 300211 China

**Keywords:** Proximal humerus, Bone tumors, Microwave, Surgery, Clinical efficacy

## Abstract

**Background:**

The present study aimed to evaluate the indications, feasibility, clinical effectiveness and complications of the treatment with microwave in situ inactivation followed by curettage and bone grafting assisted with internal fixation, for the proximal humerus tumors.

**Methods:**

The clinical data of 49 patients with primary or metastatic tumor of the proximal humerus who received intraoperative microwave inactivation in situ with curettage and bone grafting in our hospital from May 2008 to April 2021 were retrospectively analyzed.

**Results:**

There were 25 males and 24 females, with an average age of 57.6 ± 19.9 years (range, 20–81). All patients were followed up for 7 to 146 months, with an average period of 69.2 ± 39.8 months. Up to the last follow-up, 14 patients died. The 5-year overall survival was 67.3%, and 5-year tumor-specific survival was 71.4%. The 5-year tumor-specific survival rates were 100% for aggressive benign tumors or low potential malignancy tumors, 70.1% for primary malignancies, and 36.9% for metastatic tumors. The average preoperative MSTS, constant-Murley and VAS scores were 16.81 ± 3.85, 62.71 ± 12.56 and 6.75 ± 2.47, which were all significantly improved at 6 weeks after operation and at the final follow-up (*P* < 0.05).

**Conclusions:**

Microwave inactivation in situ and curettage and bone grafting are a feasible treatment for tumors of proximal humeral, especially for malignant tumors and metastases, without the necessity of the replacement of the shoulder, with little trauma and good upper limb function, and with low local recurrence and distant metastasis.

## Background

Following the location around knee, the proximal humerus is the second predilection location for all primary or metastatic tumors [1]. With the result of the combined understanding of the biology and staging of tumors, simultaneous improvements in reconstructive techniques, and the development of effective adjuvant chemotherapy, it is usually treated with limb-sparing techniques [2]. However, limb-salvage techniques for malignant tumors involving the proximal humerus pose reconstructive challenges in terms of curative resecting an adequate amount of the tissue for oncologic purposes and preserving enough tissues for functional purposes. Numerous reconstruction modalities with its own set of pros and cons have been advocated for the reconstruction of the defects after limb salvage resection, including the use of vascularized fibular autograft [3], allograft arthrodesis [4], osteoarticular allografts [5], and prosthetic replacements [6], allograft-prosthesis composite (APC) [7]. The major obstacle following a limb-sparing resection of the proximal humerus is the restoration of shoulder girdle stability. However, in order to obtain the maximize local control of the tumors, the stabilizing structures of the shoulder such as the rotator cuff, all or part of the deltoid muscle, together with the axillary nerve are often sacrificed, which result in dysfunction of the shoulder in turn.

The microwave machine with 2450 MHz power source and cylindrical applicator was independently developed and microwave inactivation in situ technology was initially used in the management of tumors in the scapular, pelvis and other extremities in the authors’ hospital, and good clinical results were achieved since 1980. The purpose of this study is to evaluate the pilot results of the surgery and the ultimate outcomes of oncological and functional of the consecutive case series who were managed by microwave-induced hyperthermia in the treatment of tumors of the proximal humerus. We attempted to answer following questions: (1) How successful is the procedure in achieving local tumor control? (2) The tips and tricks of microwave therapy for proximal humeral tumors; (3) The function of shoulder joint after microwave inactivation in situ for different tumors in the proximal humerus; and (4) What are the complications of the procedure over time?

## Material and methods

### Study design and setting

We retrospectively reviewed the electronic and paper charts of all 106 consecutive patients who underwent microwave inactivation in situ at the authors’ institution for musculoskeletal tumors of the proximal humerus between May 2008 and August 2021. Patients demographics, clinical characteristics including tumor staging, adjuvant treatments,imaging information, surgical details, complications, oncology and functional status were recorded. This study followed the guidelines of the “Declaration of Helsinki” and informed consent was obtained from all of the participating patients.

### Participants

The microwave machine with 2450 MHz power source and cylindrical applicator was independently developed and microwave inactivation in situ technology was initially used in the management of tumors in the scapular, pelvis and other extremities in the authors’ hospital, and good clinical results were achieved since 1980. The inclusion criteria for this study were patients who underwent microwave inactivation in situ in the proximal humerus. Patients would be included following the exclusion criteria: (1) Patients with huge tumors involving important blood vessels and nerves cannot be treated with limb salvage procedures; (2) patients with primary malignant or metastatic tumors who were treated with microwave inactivation in situ and the reconstruction with prosthesis or other modalities; (3) patients with bone tumors and metastases other than the proximal humerus or patients treated with microwave inactivation in situ but with the first generation microwave machine before 2002; (4) patients with too poor conditions to tolerant the procedure or contraindications for surgery; and (5) the expected survival time of metastatic tumor is less than 6 months. Metastatic lesions and multiple myeloma were not candidates unless they had a solitary metastasis from the primary tumor and no disease elsewhere detected.

**Fig. 1:**
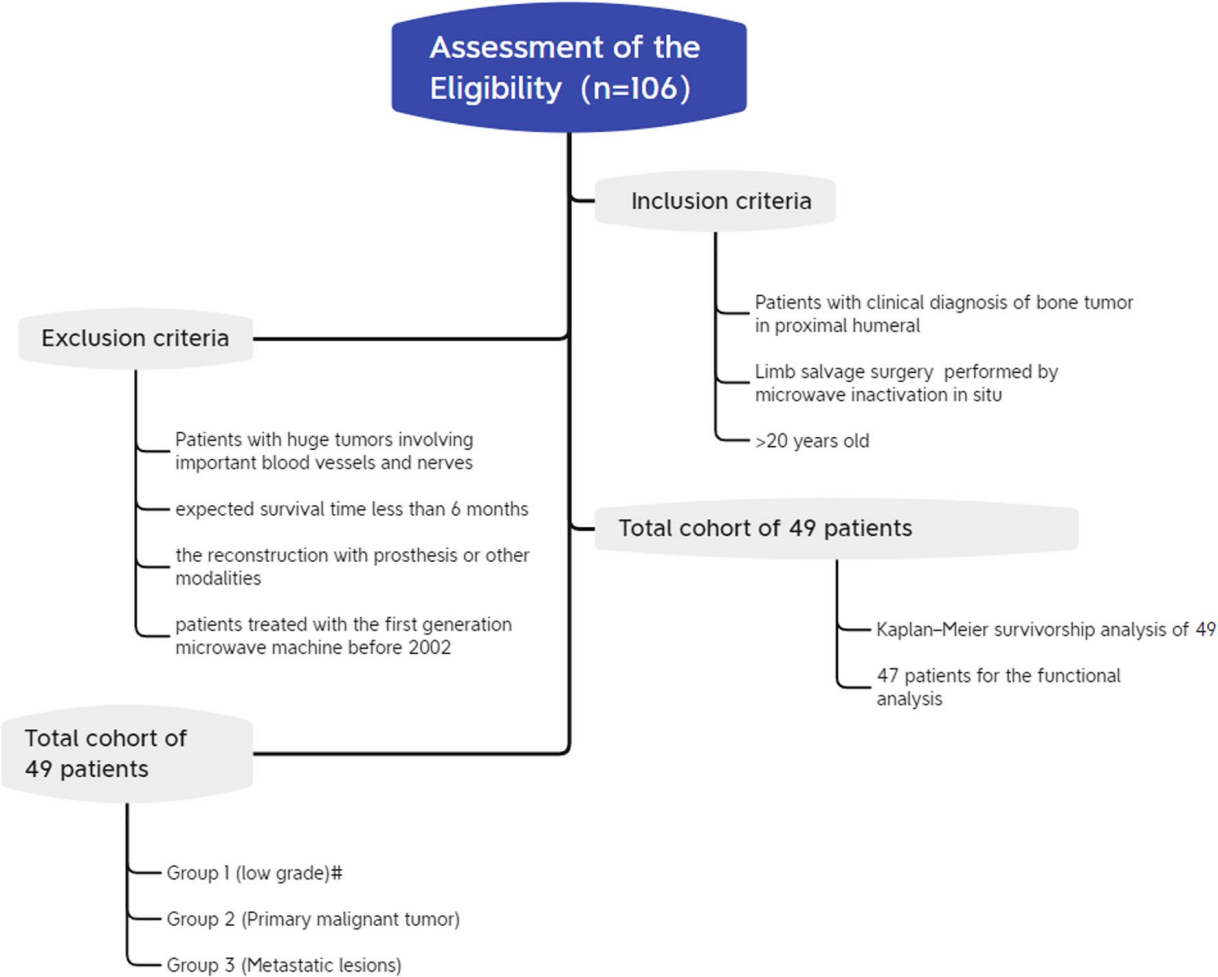
Flow diagram of patient recruitment and follow-up

According to the inclusion and exclusion criteria above, 49 patients formed the patient cohort, including 20 cases (40.8%,20/49) of aggressive benign tumor or low-grade malignant tumor, 17 cases (34.6%, 17/49) of primary malignant tumor and 12 cases (24.6%, 12/49) of metastatic tumor (Fig. 1). Thirteen patients (26.5%, 13/49) were admitted to hospital with pathological fracture, while the rest were pain accompanied by swelling in 36 patients, with an average of 7.2 months (1.5–9.2 months).

### Chemotherapy

As malignant tumors around the knee, we reported previously [8], perioperative systemic chemotherapy was performed for patients with malignant tumors, which are sensitive to chemotherapy, and two kinds of chemotherapy regimens were used: AP (Adriamycin, Cisplatin) and DIA (Cisplatin, Ifosfamide, and Adriamycin). Those with metastasis or hematologic malignancies were treated with adequate treatment of primary targeted therapy or appropriate chemotherapy as hospital protocol.

### Preoperative planning

All patients undergoing consideration for limb salvage surgery are evaluated in a systematically and individually combinatorial fashion before surgery. All patients received conventional anteroposterial-lateral shoulder radiographs and conventional 3-dimensional CT or enhanced CT and MRI examinations as a preliminary imaging method before surgery to assess the extent of tumor, bone damage and surrounding soft tissue invasion. X-ray and CT were used to evaluate the damage degree of proximal humerus cortex, MRI or enhanced MRI were used to determine the scope of extraparticular microwave therapy or intraarticular hyperthermia. Tumor diagnosis is confirmed by CT-guided needle biopsy (for patients initially coming to our facility) or by reviewing cell blocks at the referral facility. During a CT-assisted needle biopsy, the location of the trajectories must be meticulously considered as it must be excised with the tumor at the time of resection, especially for tumors without effective adjuvant treatments.

**Fig. 2:**
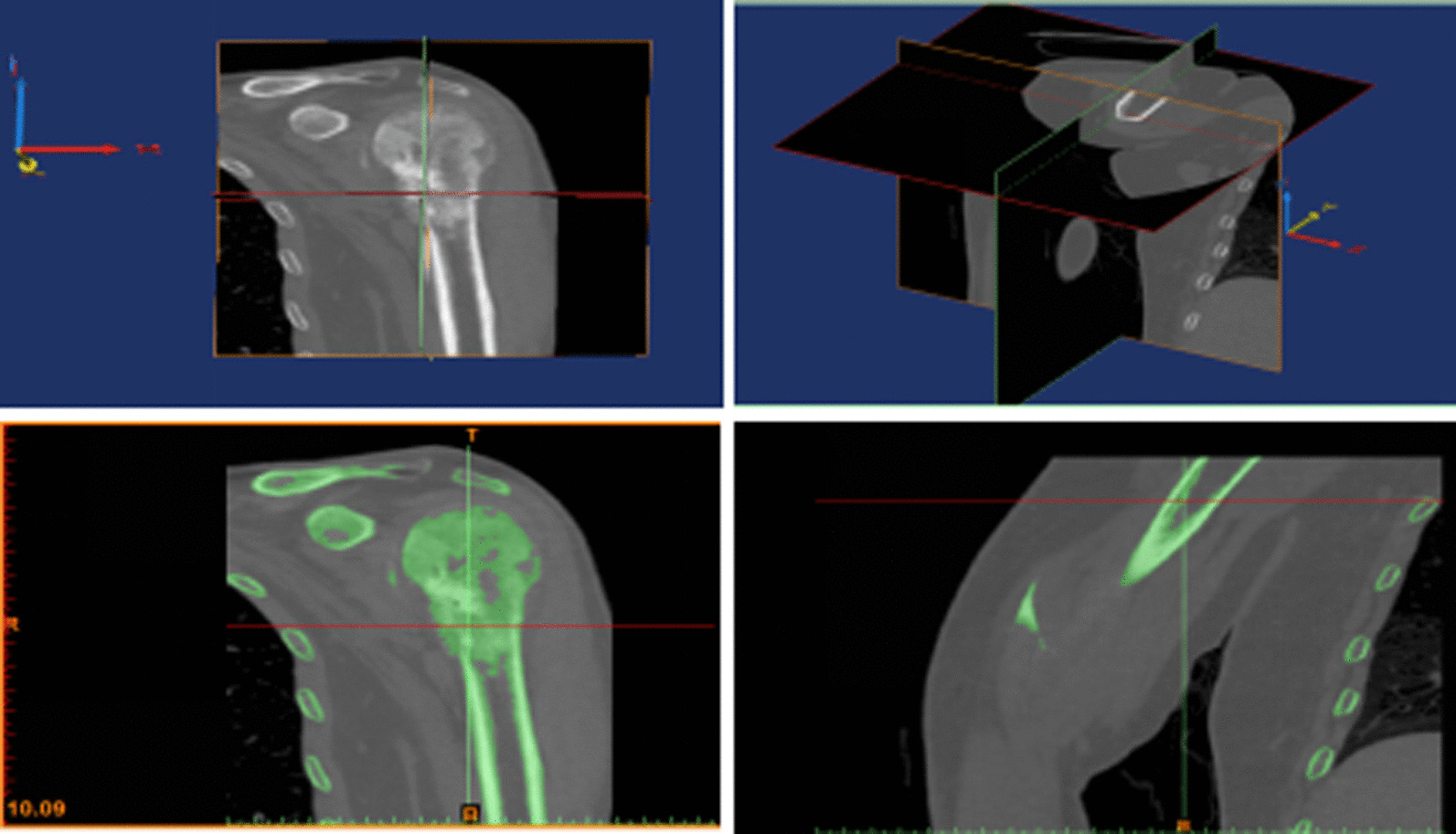
Preoperative design of primary malignancies and metastases Preoperative DICOM data from CT scan of humerus were transferred to Mimics software system (Fig. 2). The range of bone damage within the lesion was drawn by threshold segmentation. The maximum diameter of the tumor was measured by combining coronal, sagittal, and transverse data, and the location of array and the number of thermal fields were estimated, and the overlap of each potential hyperthermia range was noted

For patients with primary malignant tumor or metastatic tumor, 9 patients underwent chest CT examination in the early stage, and the remaining 25 patients underwent chest thin-sliced CT or whole body bone imaging (ECT) or PET-CT scan to evaluate the presence or absence of systemic (metastatic) disease. A full-length CT examination of the humerus was performed to determine the presence of skipping lesions. Preoperative DICOM data from CT scan of humerus were transferred to Mimics software system (Fig. 2). The range of bone damage within the lesion was drawn by threshold segmentation. The maximum diameter of the tumor was measured by combining coronal, sagittal, and transverse data, and the location of array and the number of thermal fields were estimated, and the overlap of each potential hyperthermia range was noted. Combined with preoperative MRI, the range of proximal humerus hyperthermia was determined to prevent the missing of the potential skip metastatic lesions.

Eleven patients were confirmed to have extra-articular soft tissue masses by MRI or enhanced MRI. Preoperative decision was made to first implement extra-articular microwave therapy, followed by intramedullary and bone microwave hyperthermia to minimize the risk of recurrence. All patients underwent microwave inactivation, curettage and bone grafting, and internal fixation was determined according to the nature of the tumor and the presence of pathological fractures.

### Pathology

Final determination as to surgical treatment was made on the bases of repeated imaging studies combined with needle biopsy results, and patients were divided into three treatment modalities after induction treatment. Group A: 20 aggressive benign tumors or low potential malignancy tumors (3 aneurysmal bone cyst, 7 giant cell tumor, 2 chondroblastoma, 8 chondrosarcoma in grade I); Group B: 17 primary bone sarcomas (10 osteosarcomas, 5 chondrosarcomas, 1 malignant fibrous histiocytoma and 1 Ewing's sarcoma); Group C: 12 metastatic or hematologic malignancies (Table 1). With regard to the cases of metastatic tumors or hematologic malignancies, there were 3 hematologic malignancies and 9 metastatic tumors. In terms of the site of primary malignancy included 3 from pulmonary tumors, 1 from breast, 4 from kidney, and 1 of liver origin, all of which were single metastatic tumors.

**Table 1:** Clinical epidemiology, imaging and pathological feature

Characteristics	Patients (N = 49)	(%)
Sex		
Male	25	51.0
Female	24	49.0
Age		
20–39	9	18.3
40–59	22	44.9
> 60	18	36.8
Size of tumor		
≤ 5 cm	20	40.8
> 5 cm	29	59.2
Soft tissue masses		
Present	11	22.4
Absent	38	77.6
Pathologic fracture		
Present	13	26.5
Absent	36	73.5
Histologic classification		
Group 1 (low grade)^#^		
Aneurysmal bone cyst	3	6.1
Giant cell tumor	7	14.3
Chondroblastoma	2	4.1
Chondrosarcoma of grade I	8	16.3
Group 2 (Primary malignant tumor)		
Osteosarcoma	8	16.3
Chondrosarcoma	5	10.2
Malignant fibrous histiocytoma	2	4.1
Ewing’s sarcoma	2	4.1
Group 3 (Metastatic lesions)		
Hematological origin	4	8.2
Breast	2	4.1
Kidney	2	4.1
Pulmonary metastasis	3	6.1
Alimentary tract	1	2.0
Surgical staging		
Group 1 (low grade)^#^		
3*	12	24.5
IA/IB	8	16.3
Group 2 (Primary malignant tumor)		
IIA/IIB	14	28.6
III	3	6.1

# Group 1 include aggressive benign tumor or low-grade malignant tumor.

*Stage 3 is aggressive benign tumor

In the 17 cases of primary malignant tumor group, there were 5 cases of Enneking stage IB, 4 cases of Enneking stage IIA, 6 cases of Enneking stage IIB and 2 cases of Enneking stage IIIB.

### Surgical procedures

#### Principles of surgery

(1) All operations were performed through the Henry approach into the surgical field and the cephalic vein was protected intra-operation, and the fusiform puncture site or biopsy incision was excised for patients of primary malignancies and metastasis. (2) If deltoid and rotator cuff were involved by the tumor, microwave-induced hyperthermia be given prior to the involving bone according to preoperative enhanced MRI. (3) For the patients of primary malignancies and metastasis followed the principle of extensive resection, shoulder dislocation routinely, if the soft tissue involved to minimize the risk of recurrence. (4) Whether to dislocate shoulder joint is determined by the soft tissue invasion of metastatic tumor.

#### Anesthesia and posture

The surgery was performed under general anesthesia with the patient in a beach chair position, or with shoulder pad height of 30° on the affected side and head tilted to the healthy side. All the surgeries were performed by the experienced musculoskeletal oncologists.

#### Microwave inactivation in situ

Treatment of aggressive benign tumor or low-grade malignant tumor: a part of Henry approach was used. After full exposure, 3.0-mm Schlottschner wire and 2.0-mm Kirschner wire were used to drill holes in the center of the tumor bone, and microwave needle and thermometer needle were implanted.

**Fig. 3:**
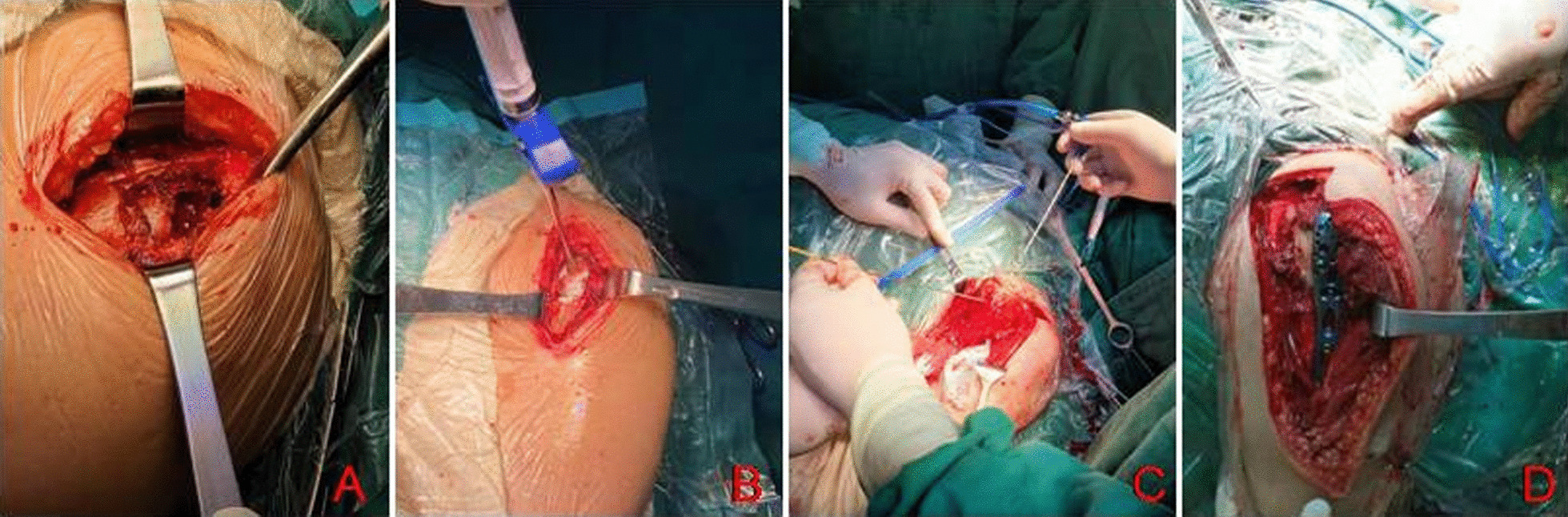
Schematic diagram of surgical operation, bone window opening for benign invasive tumor or low-grade malignant tumor (**A**); Artificial bone implantation for benign invasive tumor or low-grade malignant tumor (**B**); One patient with high-grade osteosarcoma was given microwave needle hyperthermia after shoulder dislocation during operation (**C**); Philos plate implantation after microwave hyperthermia for fracture prevention (**D**)

Start the cold circulation system of the microwave therapy machine, check the normal operation of the water cooling circulation, start the microwave power output, and heat the tumor bone from shallow to deep at the edge of the lesion according to the simulated heating site before surgery. The initial output power was 40 W, and the power was adjusted according to the real-time temperature measurement to make the temperature in the tumor > 50℃, and the microwave machine was suspended for 5–10 min. Then, a 2.0-mm Kirschner wire was used to open a 4 × 5 cm square bone window and curettage was used to remove necrotic tumor tissue from the proximal humerus and pathological examination was performed. The microwave machine was started again and the proximal and distal end of the medullary cavity was again hyperthermia. The bone around the tumor cavity was burned with electric knife, and the artificial bone was implanted successively with sandwich method (Fig. 3).

Treatment of primary malignancies: The Henry approach was used to extend the incision. If the deltoid muscle was involved before surgery, combined with the intraoperative findings, the deltoid muscle was treated with microwave and temperature needle was used to measure the temperature dynamically. The methods of hyperthermia are as follows: After the deltoid muscle and surrounding rotator cuff were exposed, shoulder joint dislocation was first performed, and then, 2450 MHz microwave therapy machine (Nanjing Yicao Company, China) was started. Microwave inactivation was performed by heating the anterior, internal and lateral fields of deltoid muscle and soft tissue with a circular radiator. Microwave output power was 50–90 W (average 70 W). At the same time, a thermometer needle is inserted into the center around the deltoid muscle to measure temperature. According to the size of the soft tissue mass and the primary and secondary conditions, the tumor internal temperature reached more than 50 ℃ for 5–20 min. The inactivation method of tumor segment bone is the same as that of aggressive benign tumor or low-grade malignant tumor, and the inactivation time is longer, and the temperature is measured dynamically. The bone around the tumor cavity was burned by electric knife, and artificial bone was implanted successively by sandwich method. All patients received PHILOS plate fixation.

Treatment of metastasis: surgical approach and incision as described above, according to the degree of soft tissue invasion to determine whether to carry out shoulder dislocation, microwave inactivation method is the same as the primary malignant tumor.

Hyperthermia is given in situ for aggressive benign tumors or low-grade malignancies, and simultaneously for primary malignancies or metastatic tumors with soft tissue involvement. Patients with aggressive benign tumors or low-grade malignancies with pathological fractures and primary malignancies and metastases were treated with PHILOS plate fixation to reduce the risk of fracture. For patients with primary malignant tumor and metastatic tumor, 18 patients received intraarticular hyperthermia and 11 patients received intraarticular and extraarticular hyperthermia according to the degree of preoperative deltoid muscle involvement.

### Postoperative rehabilitation

Postoperative intravenous antibiotics were administrated routinely to prevent infection. The aggressive benign tumor or low-grade malignant tumor was not drained after surgery, while the primary malignant tumor and metastatic tumor group were drained for 2–3 days after surgery, and the drainage tube was removed when the drainage flow was less than 30 mL/d. The next day after surgery, active functional exercise of hand, wrist and elbow joints started. In order to achieve the optimal postoperative shoulder function, the rehabilitation training began on the third day after surgery under the strict guidance of the rehabilitation instructor. The abductor splint with 30°–45° abduction of the affected limb was not removed until 4 weeks after surgery when the active movement of the shoulder initiated. All patients treated with limb salvage surgery underwent the same postoperative functional regime.

Follow-up was performed monthly for the first three months, then once every three months for the first year, and annually thereafter to assess functional and oncological outcomes. Postoperative follow-up methods included outpatient review and telephone follow-up, and the follow-up period of this study was up to August 2021.

For patients with aggressive benign tumor or low-grade malignant tumor, shoulder X-ray and chest CT should be routinely performed once a year, unless the patient has other complaints. Routine outpatient examinations for primary malignancies or metastasis included clinical examination, anteroposterior and lateral radiography of the involving shoulder, chest thin-sliced CT, and a bone scintigraphy or local ultrasonography and MRI as necessary.

### Statistical analysis

Overall survival was calculated from date of diagnosis until death from any cause, while amputation or tumor-specific death or treatment-related death was the endpoint of this study. The endpoint for all follow-up was set to August 2021, to prevent any bias caused by non-identical follow-up of patients with few or frequent appointments.

Continuous variables are presented as the mean (standard deviation, SD) and categorical variables are expressed as numbers and percentages, where applicable. The survival analysis was evaluated using the Kaplan–Meier survival curve and the differences were estimated using the log-rank test. One-way analysis of variance (ANOVA) was used to determine the significance of intergroup differences in VAS, MSTS and CMS at baseline, 6-week post-procedure and the date of last follow-up in three groups of tumors. After then, post hoc analysis was performed using SNK-q test. Statistical significance was set at *P* < 0.05. All analyses were performed with program of R language (Foundation for Statistical Computing, Vienna, Austria).

## Results

### General characteristics

There were 25 males and 24 females with mean age of 55 ± 8.55 (range, 20–81) years at the time of diagnosis. Clinical epidemiology, imaging and pathological features of the cohort are presented in Table 1. Based on combination of imaging and pathological findings, patients were subdivided into three groups: aggressive benign tumors or low-grade malignancies (group A; *n* = 20, mean age 50.7 ± 14.2 years), primary malignancies (group B; *n* = 17, mean age 50.3 ± 14.3 years) and metastasis (group C; *n* = 12, mean age 63.9 ± 15.9 years). The baseline visual analog scale (VAS) of pain ranged from 2 to 9, with an average of 6.5 ± 1.8.

**Fig. 4:**
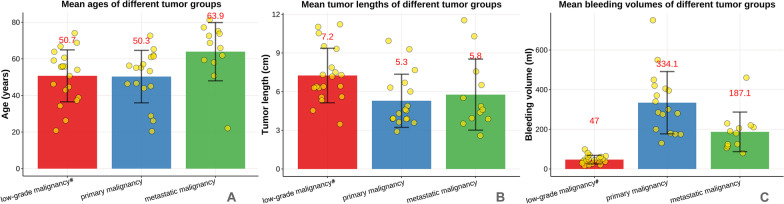
The mean age (**A**), length of tumor (**B**) and amount of intraoperative bleeding (**C**) for the three groups (i.e., the low-grade malignant tumor, primary malignancies and metastatic malignancies).

Age of diagnosis (Fig. 4A), length of tumor segment (Fig. 4B) and follow-up time between benign invasive tumor or low-grade malignant tumor group, primary malignant tumor group and metastatic tumor group were presented (Table 2). All 49 cases were followed up for 7–146 months, with an average of 69.20 ± 39.76 months. The length of tumor involvement in 49 patients was 6.2 ± 2.4 (range 2.6–11.5) cm.

**Table 2:** General results of 49 patients with bone tumors in the proximal humeral

Groups#	Pathologic fracture	Soft tissue masses	Age (y)	Size (cm)	Follow-up period (month)	Operation time (min)	Blood volume (ml)
Group 1	2	2	50.7 ± 14.2	7.3 ± 2.1	90.8 ± 28.3	57.6 ± 26.2	47.0 ± 21.6
Group 2	5	5	50.3 ± 14.3	5.3 ± 2.1	70.5 ± 40.0	188.2 ± 52.6	334.1 ± 157.0
Group 3	6	4	63.9 ± 15.9	5.8 ± 2.8	31.4 ± 27.9	140.8 ± 52.3	187.1 ± 99.7

# Group 1 include aggressive benign tumor or low-grade malignant tumor, Group 2 include primary malignant tumor, Group 3 for Metastatic lesions

All the 49 cases were operated successfully, without perioperative anesthesia or medical complications. The duration of operation was 123.3 ± 72.1 (range 30–280) min, with the intraoperative bleeding 180.9 ± 162.7 (range 15–750) ml (Fig. 4C). Intraoperative blood transfusion was performed in 2 cases in primary malignancies and 1 case in the metastasis.

### Survivorship analysis

**Fig. 5:**
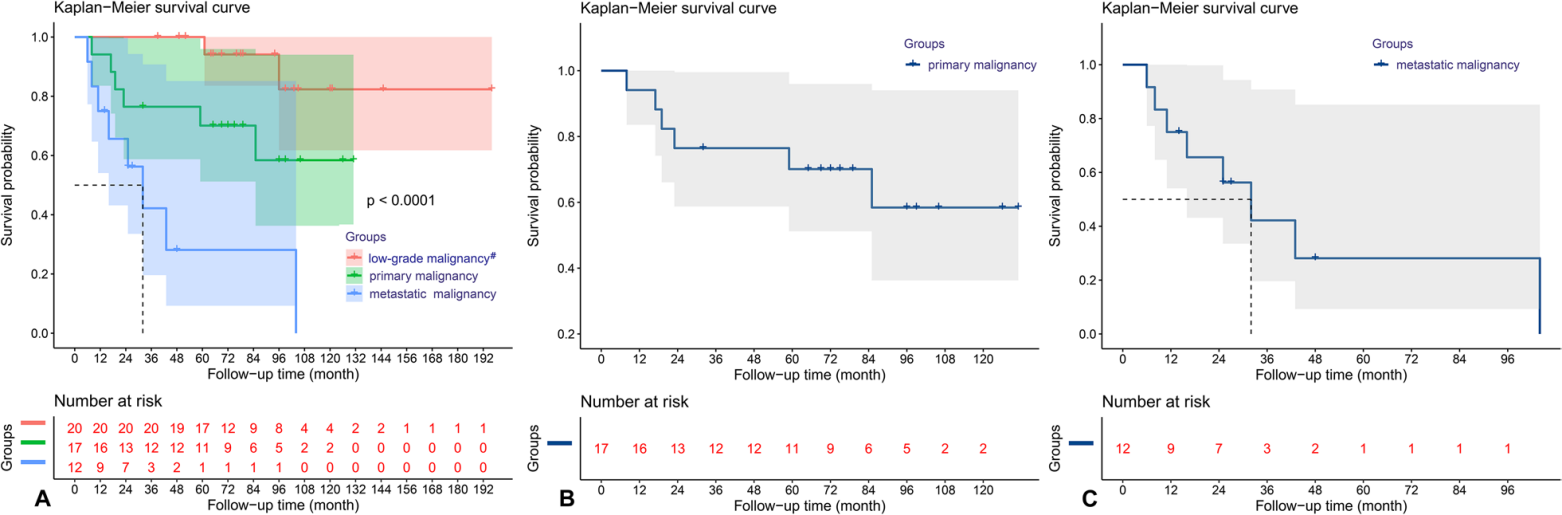
The survival analysis of the patients. The 5-year tumor-specific survival rates were 100% for benign invasive or low-grade malignancies, 70.1% for primary malignancies, and 36.9% for metastases (**A**). Kaplan–Meier survival curves for primary malignancies (**B**) and metastatic malignancies

All 49 patients were followed up for 89.5 ± 17.4 (range 6–131) months. At the last follow-up, 14 patients (28.6%, 14/49) were confirmed to have died of tumor cause, with an overall 3-year survival rate of 82.4%. 5-year tumor-specific survival rates were 100% for benign invasive or low-grade malignancies, 70.1% for primary malignancies, and 36.9% for metastatic tumors (Fig. 5). The median survival time was 120 months, 59 months and 25 months, respectively.

At the last follow-up, two patients in the benign invasive tumor group or low-grade malignant tumor group died of sudden myocardial infarction and cerebrovascular accident 94 and 120 months after surgery, respectively. Sixteen patients (55.2%, 16/29) in the primary malignant and metastatic groups survived, with overall 1-year, 3-year and 5-year survival rates of 89.0%, 50.1% and 31.3%, respectively. The 1-year, 3-year and 5-year survival rates of primary malignant tumor and metastatic tumor were 94.9%, 77.7% and 49.3%, 75.3%, 60.3% and 45.2%, respectively (Fig. 5).

Up to the last follow-up, 4 patients (8.2%, 4/49) suffered local recurrence. Among them, there was 1 case (5%, 1/20) of local recurrence (relapsed at 59 months post-operatively) occured in the aggressive tumor or low-grade malignant tumor group. This patient was giant cell tumor of bone (GCTB) complicated with pathological fracture, who underwent knee joint replacement for GCTB before admission. The recurrence lesion was controlled steadily by microwave hyperthermia again. The patient developed multiple metastases to the ilium and lungs 93 months postoperatively, resulting in survival with tumor. In the primary malignancy group, there were 2 patients (11.8%, 2/17), including 1 Ewing’s sarcoma patient and 1 high-grade osteosarcoma patient, 17 and 61 months after surgery, respectively. One patient underwent shoulder arthrotomy, and the other patient developed multiple metastases, resulting in death. In the metastatic group, 1 patient (8.3%, 1/12) with small cell lung cancer recurred 11 months after surgery with multiple metastases and eventually died. There was no significant difference in local recurrence rate among the three groups (all *P* > 0.05).

### Imaging evaluation

**Fig. 6:**
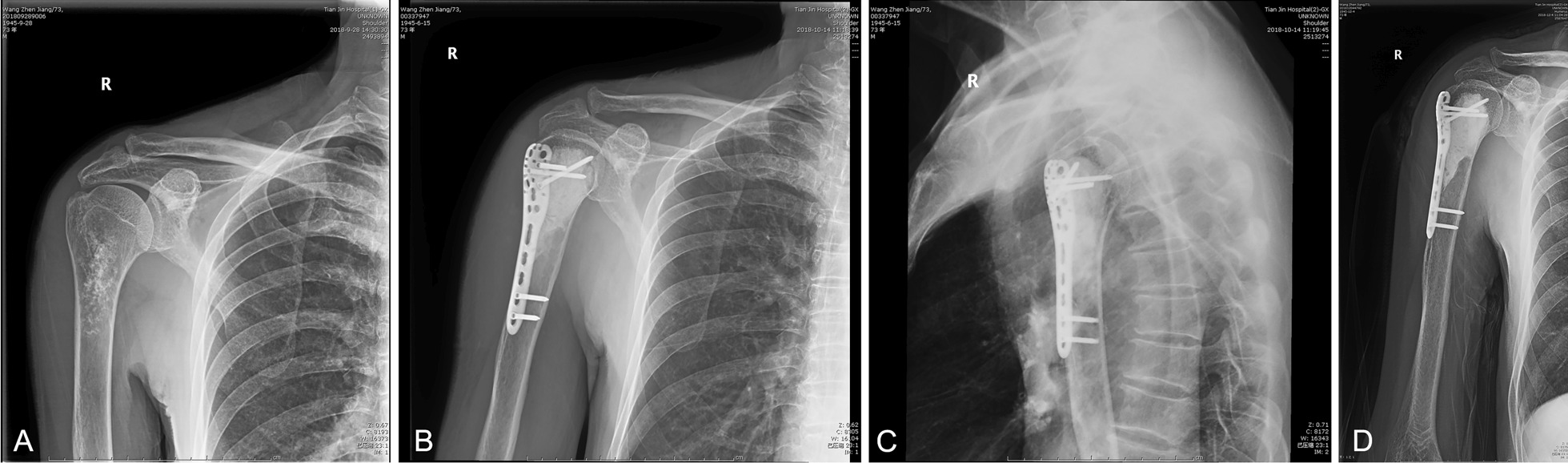
A 73-year-old male with enchondroma on the proximal humerus, who was operated with tumor resection followed by microwave-induced hyperthermia in situ and internal fixation with bone cement. **A**: anteroposterior (AP) radiograph of the right shoulder showed sand-like calcification lesion of the upper humerus. **B** & **C**: the AP and lateral radiographs of the right shoulder at 3 days after operation. **D**: the AP radiograph of the right shoulder at 2 months after operation

The osteogenesis of shoulder after artificial bone implantation was evaluated by X-ray film, and no subluxation or dislocation of shoulder was found (see the case presented in Fig. 6). Up to the last follow-up, in 1 patient, there were still some regional lucency lines at the allogenic bone–host bone interface, and bone resorption could be seen near the lucency line. All 5 patients had bone resorption of grafted or inactivated bone to varying degrees. Early follow-up X-ray showed bone resorption at the graft-host bone interface, followed by greater tubercles and lower BMD in the inner and outer cortical areas.

### Functional outcome

At the final follow-up, 1 patient in the primary malignant tumor group had amputation and 1 patient had poor range of motion of shoulder, while the other patients had no significant impact on their daily life and work and all obtained satisfactory shoulder function.

The range of motion of shoulder joint in all patients increased significantly after operation. At the last follow-up, the improvement range of shoulder flexion and upward lift, lateral external rotation, internal rotation and abduction were 142.0 ± 26.8, 51.7 ± 11.9, 59.0 ± 13.6 and 139.4 ± 28.9, respectively. Compared with preoperative 110.2 ± 35.5, 35.7 ± 14.9, 50.0 ± 15.7, 109.1 ± 38.5, the differences were statistically significant (*t* = − 4.95, *P* < 0.05; *T* = − 5.81, *P* < 0.05; *T* = − 3.02, *P* < 0.05; *T* = − 4.36, *P* < 0.05).

Preoperative ROM of benign invasive tumor or low-grade malignant tumor group, primary malignant tumor group and metastatic tumor group were 110.2 ± 35.5, 35.7 ± 14.9, 50.0 ± 15.7, 109.1 ± 38.5, respectively. At the last follow-up, they were 142.0 ± 26.8, 51.7 ± 11.9, 59.0 ± 13.6, and 139.4 ± 28.9, respectively, which were higher than those after operation, and the differences were statistically significant (*P* < 0.001).

**Fig. 7:**
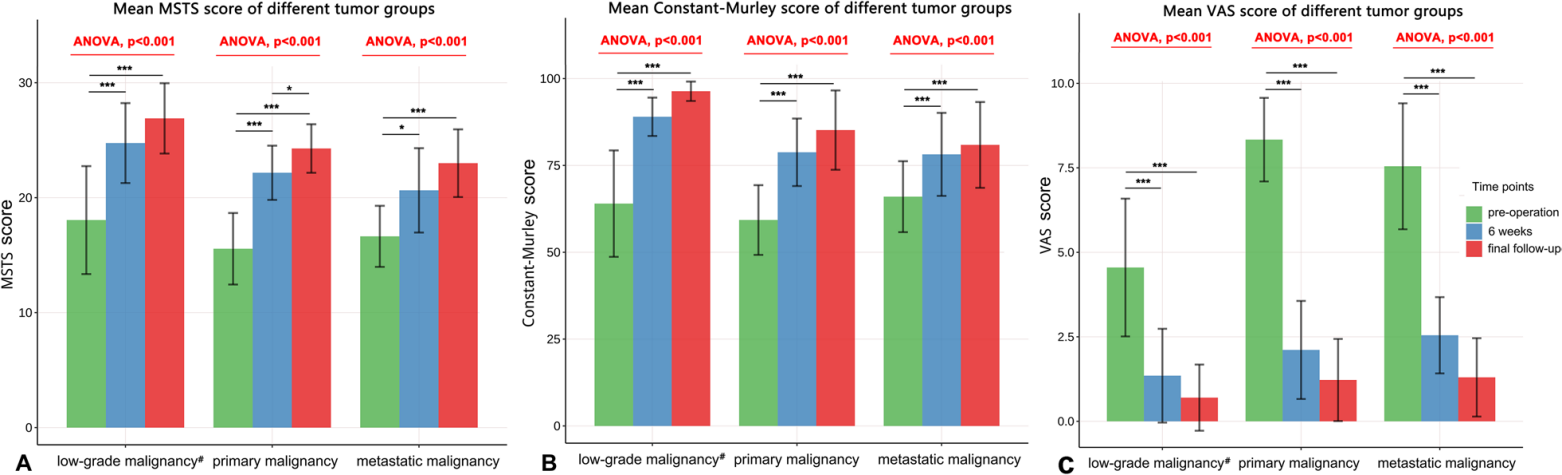
Mean MSTS score (**A**), Constant-Murley score (**B**) and VAS score (**C**) of the three tumor groups at different follow-up times. The results of one-way ANOVA showed that MSTS, Constant-Murley scores and VAS were significantly improved in the three groups after surgery (*P* < 0.001). The bars and error lines in the figure represent mean ± standard error (SD). *p < 0.05; **p < 0.01; ***p < 0.001

In the primary malignant tumor group, 15 patients (1 amputation) participated in the function score during the last follow-up, and all the other patients participated in the function evaluation. The MSTS score of this group was (16.81 ± 3.85) points before surgery, (22.87 ± 3.51) points 6 weeks after surgery, and (25.10 ± 3.13) points at the last follow-up, compared with that before surgery 6 weeks after surgery (*t* = − 8.04, *P* < 0.01) and that before surgery 6 weeks after surgery (*t* = − 11.55, *P* < 0.01). The differences were statistically significant. (Fig. 7).

MSTS scores of benign invasive tumor or low-grade malignant tumor, primary malignant tumor and metastatic tumor before operation were 18.0 ± 4.8, 15.7 ± 3.0 and 16.0 ± 3.4, respectively. 6 weeks after operation and the last follow-up were 24.8 ± 3.5, 22.2 ± 2.4, 20.6 ± 3.7 and 26.9 ± 3.0, 24.2 ± 2.2, 23.0 ± 3.0, respectively. The results of one-way ANOVA showed that the MSTS scores of patients in the three groups were significantly improved 6 weeks after surgery and at the last follow-up compared with those before surgery (*P* < 0.001). (Fig. 7).

Constant-Murley function score was (62.71 ± 12.56) score before surgery, (83.4 ± 9.32) score at 6 weeks after surgery, and (89.95 ± 9.02) score at the last follow-up. Preoperative and postoperative 6 weeks (*t* = − 9.13, *P* < 0.01), and there were statistically significant differences between preoperative and last follow-up (*t* = − 12.16, *P* < 0.01) (Table 3 and Fig. 7).

**Table 3:** Comparison of range of motion and functional score of shoulder between preoperative and last follow-up in 49 patients (X ± S)

variable	MSTS	ROM(°)				Constant-Murley
		Anteversion	ERO	IRO	Abduction	
Pre	16.8 ± 3.9	110.2 ± 35.5	35.7 ± 14.9	50.0 ± 15.7	109.1 ± 38.5	62.8 ± 12.7
LSp	25.1 ± 3.1	142.0 ± 26.8	51.7 ± 11.9	59.0 ± 13.6	139.4 ± 28.9	89.9 ± 8.9
t	− 11.5	− 4.95	− 5.81	− 3.02	− 4.36	− 12.10
P	< 0.05	< 0.05	< 0.05	0.03	< 0.05	< 0.05

*Pre* preoperatively, *LSp* last follow-up postoperatively, *VAS* visual analog scale, *MSTS* Musculoskeletal Tumor Society, *ROM* range of motion of the shoulder, *ERO* active external rotation, *IRO* active internal rotation

In this group, shoulder pain symptoms were significantly improved 6 weeks after surgery and at the last follow-up, with VAS scores of (1.44 ± 1.42°) and (0.76 ± 1.06) points, compared with preoperative (6.75 ± 2.47) points, the differences were statistically significant (*T* = − 13.05, *T* = 15.6; *P* < 0.01, *P* < 0.01). (Fig. 7).

VAS scores of patients in the benign invasive tumor group or low-grade malignant tumor group, primary malignant tumor group and metastatic tumor group 6 weeks after surgery and at the last follow-up are shown in Fig. 7.

### Complications

One case of Ewing's sarcoma recurred 6 months after surgery and underwent shoulder arthroplasty. One patient with osteosarcoma could not tolerate vomiting caused by postoperative chemotherapy, and the reexamination was good 3 months after internal fixation, but the patient refused to continue chemotherapy and continued dynamic observation. One patient developed shoulder abduction failure, which may be related to axillary nerve injury resulting in deltoid weakness.

**Fig. 8:**
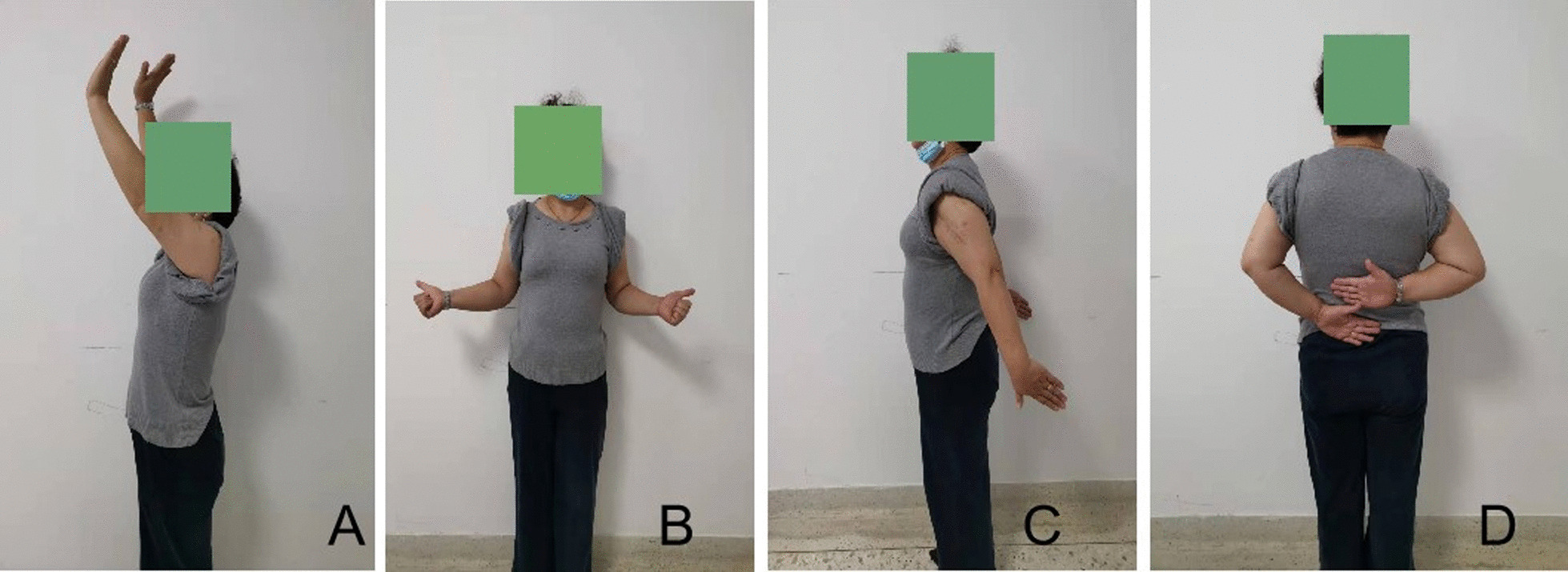
A 45-year-old female patient with osteosarcoma underwent MWA-in situ following internal fixation of Philos in the right proximal humerus. *Notes*: **A**–**D** at the last follow-up, the patient recovered satisfactory contour and function of the shoulder

One case had bone cyst complicated with pathological fracture and wrist dorsiflexion weakness after surgery, which was improved 6 months after surgery. Considering the irritation of internal fixation, internal fixation was removed and radial nerve exploration was performed 1 year after surgery, and the symptoms of the patient disappeared completely after surgery (Fig. 8).

## Discussion

### *Mechanism and advantages of microwave *in situ* inactivation*

Thanks to the joint efforts of multidisciplinary teams, the life expectancy of patients with malignant tumors has significantly improved, and limb salvage therapy has replaced amputation as the prevailing limb tumor treatment. However, there is no consensus on the treatment of proximal humeral bone tumors [9]. There are various methods to reconstruct bone following limb bone tumor resections, such as autologous bone transplantation, in vitro inactivation of autologous tumor cells (e.g., high-temperature inactivation, alcohol immersion, in vitro radiotherapy), replantation, allotransplantation, and artificial full-length prosthesis replacement. However, each reconstruction method has its shortcomings [10–12]. Further research is needed to understand how to achieve satisfactory shoulder function without sacrificing tumor control.

Tumor cells have poor blood supply, and their metabolism is mainly anaerobic. Their microenvironment is acidic, and they are more sensitive to heat than normal cells. After heating, the synthesis of DNA, RNA, and protein in malignant tumor cells is inhibited, the permeability of the cell membrane and various functions of biofilms are changed, and the activity of cell lysosomes is increased, leading to the destruction and death of cells. This is the basic molecular mechanism of hyperthermia for tumor treatment.

Microwave ablation is one method of tumor hyperthermia therapy. Its basic principle is to use the thermal effect of a microwave electromagnetic field to produce a series of inactivating effects on tumors, including direct killing [13], induction of apoptosis [14, 15], destruction of tumor blood vessels, [16] and promotion of immunity [17]. Since bone tissue is mainly composed of collagen and inorganic salts, which can withstand higher temperatures while maintaining biomechanical strength, microwave ablation has unique advantages and characteristics in the treatment of bone tumors [18].

### Oncology results and functional evaluation of patients after microwave hyperthermia

In general, relatively thorough surgery is needed for primary malignant tumors, and extensive resection positively affects local control. Due to the limited space range of the upper limbs, surgery is usually relatively safe when the tumor is large [19]. In this study, patients with primary high-grade sarcoma received neoadjuvant chemotherapy before surgery, which provided a systemic guarantee for the safety of limb salvage. Patients with metastatic tumors received systemic medical treatment and targeted therapy for primary tumors before surgery. For low-grade malignant tumors, safe surgical boundary resection is crucial to the local control rate. Microwave hyperthermia has unique advantages in the local control of tumors. All patients in this group, regardless of tumor type, were inactivated by microwave, which is safer. Up to the most recent follow-up, 16 patients (55.2%, 16/29) in the primary malignant tumor or metastatic tumor group were still alive, with overall 1-year, 3-year and 5-year survival rates of 89.0%, 50.1% and 31.3%, respectively. The 1-year, 3-year and 5-year survival rates of primary cancer and metastatic cancer were 94.9%, 77.7%, and 49.3%, and 75.3%, 60.3% and 45.2%, respectively. In this study, the patients with malignant tumors were older, with an average age of 54.4 years (20–77 years). In addition, there were 12 patients with metastatic tumors in malignant tumors, which further impacted the survival rate.

Up to the most recent follow-up of this study, 4 of the 49 patients had recurrences, including 1 case (5%, 1/20) of local recurrence in the benign invasive tumor or low-grade malignant tumor group and 1 case of preoperative giant cell tumor of bone complicated with pathological fracture. In the primary malignancy group, there were 2 patients (11.8%, 2/17) with recurrences, including 1 Ewing sarcoma patient and 1 high-grade osteosarcoma patient, 17 and 61 months after surgery, respectively. One patient underwent shoulder arthrotomy, and the other developed multiple metastases, resulting in death. In the metastatic group, 1 patient (8.3%, 1/12) with small cell lung cancer recurred 11 months after surgery with multiple metastases and eventually died. There was no significant difference in the local recurrence rate among the three groups (all *P* > 0.05). The low recurrence rate in this group was considered to be related to the following factors: Priority was given to tumors during surgery; intraoperative hyperthermia was only given to benign invasive tumors or low-grade malignant tumors; hyperthermia was also given to primary malignant tumors or metastatic tumors if soft tissue was involved; for highly malignant tumors, preoperative adjuvant chemotherapy and other related medical treatments were used, local swelling subsided, and reaction area shrunk, further improving the local safety with the help of microwave ablation.

Treating proximal humeral tumors is challenging [20, 21] for several reasons: Maintenance of good shoulder function depends on several important factors, such as the rotator cuff muscles (especially the deltoid) and the axillary nerve. Because the axillary nerve is located on the lower surface of the deltoid muscle, it is often injured during tumor resection. When treating proximal humeral malignancies, especially primary malignancies, routine removal of the deltoid muscle and axillary nerve is recommended by some authors to achieve a safe surgical boundary at the expense of shoulder function [22]. The rotator cuff plays an important role in the stability of the shoulder joint, and partial rotator cuff resection is sometimes required to achieve broad surgical boundaries at the expense of shoulder function. In this study, the deltoid muscle (part or all), axillary nerve, and rotator cuff were completely preserved thanks to the auxiliary effect of microwaves, which is the basis for obtaining good shoulder joint function in this study.

In this study, microwave in situ inactivation achieved better functional improvement than traditional reconstruction methods. At the last follow-up visit, MSTS scores and Constant-Murley scores of the shoulder joints in this group were 25.1 and 89.9 points on average, significantly improved from pre-operation scores, and the difference was statistically significant. At the last follow-up, the average shoulder active abduction was 139.4°, and the average shoulder active flexion was 142°. Of course, there are some shortcomings in this study. In the primary malignant tumor group, the shoulder abduction function was slightly worse, which may be related to the dislocation of the shoulder joint during the operation to completely inactivate the tumor. In addition, the deltoid muscle was partially resected during the operation in some patients.

In addition, due to the thermal coagulation effect of microwaves, tissue coagulation in tumors can reduce the amount of intraoperative blood loss and shorten the operation time. Among the 49 patients in this group, the average amount of surgical bleeding in the 3 groups was 55.0, 325 and 185 ml, respectively, and the operation time was saved.

### *Microwave inactivation for *in situ* treatment of proximal humeral tumor notes*

The incidence of intramedullary jumping lesions in osteosarcoma is 1.4–10% [23, 24], and their occurrence often indicates a poor prognosis. The small intramedullary jumping lesion of osteosarcoma with mild or no obvious damage to the surrounding cortex is an ideal indication for microwave ablation. In this group, the single intramedullary jumping lesion of osteosarcoma was directly ablated by microwave ablation through the medullary cavity, further improving safety. Therefore, in this study, all patients received preoperative X-ray, humerus length CT, MRI or enhanced MRI to take "series,” and in preoperative biopsies, tumor blood source conventional isotope scanning confirmed the presence of skipping lesions. We believe that the main points of inactivation of proximal humerus bone tumors are as follows: ① Due to the difference in tumor composition among the three groups, multipoint inactivation and real-time continuous temperature measurement are used to ensure that the inactivation temperature is reached. In addition, the inactivation of patients in the three groups should be "individualized," and the inactivation time of patients in the primary malignant tumor and metastatic tumor groups should be appropriately extended to ensure efficacy. ② There are many neoplastic vessels in tumor lesions, such as giant cell tumors of the proximal humerus, aneurysmal bone cysts, and bone metastasis of renal cancer. Microwave ablation pretreatment should be used before curettage to reduce tumor bleeding. ③ Microwave in situ inactivation in the treatment of primary malignant bone tumors or metastatic tumors of the proximal humerus should be assisted with plate treatment to prevent or treat fractures. (4) MRI or enhanced MRI is more accurate in determining the tumor segment range of the proximal humeral tumor to achieve an accurate inactivation range and prevent residue. (5) Due to the consideration of axillary nerve and vascular factors, vessels and nerves should be identified and protected prior to initiating heat therapy. In this study, the heat insulation effect of copper mesh and the cooling effect of low-temperature physiological saline were used to protect the surrounding soft tissues, blood vessels and nerves.

### Limitations of this study

This study has the following limitations: (1) It was a retrospective study with a small sample size and no control group. (2) The time span of the cases was large, and the microwave used for therapy was replaced during the study, but the microwave power and basic parameters of all patients remained unchanged. (3) Compared with the distal femur and proximal tibia, the incidence of proximal humeral bone tumors is low, and the sample size is small due to the strict control of indications in this group. If a larger case sample can be accumulated, the data will be more convincing. In summary, the present results suggest that microwave inactivation, curettage, and bone grafting are safe and effective for proximal humeral tumors, not only for benign invasive tumors and low-grade proximal humeral bone tumors but also for patients with primary malignant bone tumors and metastases. Further investigation is needed to determine whether this method is suitable for tumors with extensive soft tissue infiltration. In view of this, the research team will continue to expand the case sample to increase the persuasion; follow-up of all postoperative cases will continue to be extended to provide a more detailed and objective evaluation of oncology and function.

## Conclusions

This group of patients is small but valuable because the deltoid muscle and rotator cuff can be preserved with chemotherapy and adjuvant therapy combined with microwave therapy, inactivation of the primary lesion and its surrounding area, and curettage and bone grafting of the lesion. Microwave inactivation curettage and bone grafting is a safe, feasible and reliable option for the treatment of proximal humeral tumors without joint replacement.

## Data Availability

The datasets used and/or analyzed during the current study are available from the corresponding author on reasonable request.

## Abbreviations

SD: Standard deviation
ANOVA: Analysis of variance
VAS: Visual analog scale
ROM: Range of motion
MSTS: Musculoskeletal Tumor Society
